# CETP Expression Protects Female Mice from Obesity-Induced Decline in Exercise Capacity

**DOI:** 10.1371/journal.pone.0136915

**Published:** 2015-08-27

**Authors:** David A. Cappel, Louise Lantier, Brian T. Palmisano, David H. Wasserman, John M. Stafford

**Affiliations:** 1 VA Tennessee Valley Healthcare System, Nashville, Tennessee, United States of America; 2 Division of Diabetes, Endocrinology, & Metabolism, Department of Internal Medicine, Vanderbilt University School of Medicine, Nashville, Tennessee, United States of America; 3 Department of Molecular Physiology and Biophysics, Vanderbilt University School of Medicine, Nashville, Tennessee, United States of America; National Cancer Institute, UNITED STATES

## Abstract

Pharmacological approaches to reduce obesity have not resulted in dramatic reductions in the risk of coronary heart disease (CHD). Exercise, in contrast, reduces CHD risk even in the setting of obesity. Cholesteryl Ester Transfer Protein (CETP) is a lipid transfer protein that shuttles lipids between serum lipoproteins and tissues. There are sexual-dimorphisms in the effects of CETP in humans. Mice naturally lack CETP, but we previously reported that transgenic expression of CETP increases muscle glycolysis in fasting and protects against insulin resistance with high-fat diet (HFD) feeding in female but not male mice. Since glycolysis provides an important energy source for working muscle, we aimed to define if CETP expression protects against the decline in exercise capacity associated with obesity. We measured exercise capacity in female mice that were fed a chow diet and then switched to a HFD. There was no difference in exercise capacity between lean, chow-fed CETP female mice and their non-transgenic littermates. Female CETP transgenic mice were relatively protected against the decline in exercise capacity caused by obesity compared to WT. Despite gaining similar fat mass after 6 weeks of HFD-feeding, female CETP mice showed a nearly two-fold increase in run distance compared to WT. After an additional 6 weeks of HFD-feeding, mice were subjected to a final exercise bout and muscle mitochondria were isolated. We found that improved exercise capacity in CETP mice corresponded with increased muscle mitochondrial oxidative capacity, and increased expression of peroxisome proliferator-activated receptor gamma coactivator 1-alpha (PGC-1α). These results suggest that CETP can protect against the obesity-induced impairment in exercise capacity and may be a target to improve exercise capacity in the context of obesity.

## Introduction

Beyond its role in weight loss, exercise is critical to overall health, as impaired exercise capacity correlates with increased risk of death from all causes [1], and impaired exercise capacity has a stronger correlation with mortality than increased BMI among men with diabetes [2]. Pharmacological approaches to reduce obesity have been limited by high attrition rates and unclear or even harmful effects on cardiovascular health [3, 4]. Obesity reduces exercise tolerance, making adherence to exercise regimens more difficult [5].

The ability of skeletal muscle to oxidize fuel substrates is crucial to exercise capacity. Reduced muscle glucose oxidation and oxidative enzyme activity [6] is observed in individuals with impaired glucose tolerance and diabetes [7]. Therefore, changes in skeletal muscle oxidative capacity likely contribute to the obesity-related decline in exercise capacity. Reciprocally, activation of exercise pathways leads to a reduction in obesity-related complications including diabetes and cardiovascular risk [1, 5, 8, 9].

Our previous studies have suggested that transgenic expression of cholesteryl ester transfer protein (CETP) in mice may protect against obesity-related declines in exercise capacity [10]. CETP is a lipid transfer protein that facilitates the flux of lipids between serum lipoproteins including HDL and VLDL [11, 12]. CETP is expressed in humans and primates but is not naturally expressed in mice [13]. We observed that transgenic expression of CETP in female mice was associated with increased muscle glycolysis during fasting and protection against diet-induced insulin resistance [10]. CETP did not increase glycolysis or promote insulin sensitivity in males. Because the CETP-expressing mice showed increased muscle glycolysis, we propose that CETP-expressing female mice might have improved capacity of muscle to oxidize glucose and increased exercise tolerance when fed high fat diet (HFD).

Studies in humans have suggested that CETP may improve cholesterol efflux and glucose metabolism in a sexually dimorphic manner. Serum from females with high CETP activity showed increased ability to take up cholesterol from macrophages compared to serum from females with low CETP activity [14]. Similar experiments performed by the same group did not show a correlation between CETP activity and cholesterol uptake from macrophages [14]. Additionally, studies of obese women treated with gastric bypass surgery showed an inverse correlation between changes in CETP mass and glycemia and HOMA-IR score [15]. These observations in humans coupled with our findings that CETP expressing female but not male mice had increased glucose oxidative capacity led us to focus our studies on the female mice.

To test the hypothesis that CETP expression protects female mice against HFD-induced decline in exercise capacity, we measured exercise capacity in CETP-expressing female mice compared to wild-type non-transgenic female littermates (WT) over the course of HFD-feeding. Importantly, mice were not exercised between each test, as we were interested in studying intrinsic exercise capacity and not response to exercise training. Additionally, our goal was not to induce weight loss through exercise but to understand the metabolic changes that might improve exercise capacity in the context of obesity. While there was no difference in exercise capacity on chow diet, CETP-expressing mice were protected from the obesity-related decline in exercise capacity. This improvement in exercise capacity corresponded with an increase in mitochondrial oxidation of glutamate/malate substrates.

## Materials and Methods

### Animals and Diets

Mice expressing a simian CETP gene under control of a constitutive promoter on a C57B/6 background were purchased from Jackson Laboratories (C57BL/6-Tg(CETP)1Pnu/J, Stock Number: 001929) [11, 12]. Because of our previous observations that female CETP mice show increased glycolysis [10] and the sexual dimorphism in CETP’s effect on metabolism in humans [14, 15], only female mice were included in the study. At 12 weeks of age, animals were placed on a high fat diet consisting of 60% fat from lard and carbohydrate content comprised of cornstarch (Research Diets D08060104) as previously described [10]. Body composition was determined using a mq10 NMR analyzer (Bruker Optics) located at the Vanderbilt Mouse Metabolic Phenotyping Center. All procedures were performed in accordance with National Institutes of Health Guidelines for the Care and Use of animals and approved by the Institutional Animal Care and Use Committee at Vanderbilt University.

Euthanasia of mice was performed by carbon dioxide overdose or cervical dislocation under anesthesia. Death was ensured by secondary physical method.

### Exercise Studies

Animals in this study were subject to an exercise tolerance test every 2 weeks. Exercise testing was performed as previously described [16]. Briefly, animals were acclimated to treadmill running with a 10-minute run at a constant speed of 10m/min for 5 days prior to the first exercise tolerance test. The initial trial was performed on chow-fed mice, before the HFD-feeding was begun. Mice were placed on an enclosed treadmill in which oxygen was sampled allowing for the measurement of whole body oxygen uptake. Mice were run at an initial speed of 10 m/min. Every 3 minutes speed was increased by 4 m/min. Mice were encouraged to run with an electrical shock grid at the back of the treadmill (1.5 mA, 200 ms pulses, 4 Hz). The study was continued until the animal was exhausted as defined by remaining on the electrified grid for 5 seconds. The experimenter was blinded as to the genotype of the animal during the exercise test. Mice were housed in standard cages with no access to running wheels between exercise testing sessions. In the final trial, mice were run at a constant speed of 10 m/min for 15 minutes after 12 weeks of HFD-feeding. Following the exercise study, animals were sacrificed and tissues were collected and flash frozen.

### Mitochondrial function analysis

Mitochondrial oxidative capacity was measured by oxygen consumption of e*x-vivo* muscle fibers isolated from the red gastrocnemius muscle using an Oroboros Oxygraph2k system as previously described [17, 18]. Muscle fibers were collected immediately following the final constant speed run and then immediately placed into a solution consisting of ethylene glycol tetra acetic acid (EGTA)-calcium buffer (2.7 mM), imidazole (20 mM), taurine (20mM), K+ /4 morpholinoethanesulfonic acid (K-MES; 50 mM), MgCl_2_ (6.56 mM), Na_2_ATP (5.77 mM), and phosphocreatine (15 mM), pH 7.1. The muscle fibers bundles were separated with sharp forceps to maximize surface area exposure and then incubated in the above solution containing 50 μg/mL saponin for 30 minutes to maximize permeabilization. Fibers were then transferred into the Oxygraph2k Respirometer for analysis. The wells of the respirometer were held at 22C and filled with a solution consisting of: KCl (30 mM), K-MES (105 mM) K_2_HPO_4_ (10 mM), MgCl_2_-6H_2_O (5mM), EGTA (1mM), and bovine serum albumin (0.5g/L), pH 7.1. Before oxygen measurement, the chamber was hyper-oxygenated to 250 nmol/mL by injecting 100% O_2._ Following oxygen consumption measurement, muscle fibers were desiccated and dry weight was measured.

To measure muscle substrate utilization, two separate protocols were used on the isolated muscle fibers. The first protocol measured oxygen consumption from mitochondrial complex-I linked substrates (10mM glutamate and 2 mM malate) in the presence of 2mM ADP. The second protocol measured oxygen consumption from fatty acid derived substrate (75 μM palmitylcarnitine) in the presence of 2mM malate and 2mM ADP.

### Gene Expression

RT-PCR was used to measure gene expression. RNA was extracted from snap frozen muscle collected following the final exercise test done at 12 weeks (Trizol, Life Technologies). cDNAs were synthesized using 1μg RNA template (iScript cDNA synthesis kit, BioRad). RT-PCR was conducted using SYBR Green JumpStart Taq ReadyMix (Sigma) in a 20μl reaction with 400 nM primer concentration. 50 cycles of 95°C for 10 s, 58°C for 45 s, and 72°C for 60 s were performed to amplify mRNA (MyIQ, Bio Rad). Ct values were analyzed using the efficiency corrected Pfaffl method and were normalized to actin. Fold change was determined relative to WT littermates. Primer sequences were as follows:

PDK forward primer (GCATTTCTACTCGGATGCTCATG).

PDK reverse primer (CCAATGTGGCTTGGGTTTCC).

PGC1 alpha forward primer (CGATCACCATATTCCAGGTCAAG).

PGC1 alpha reverse primer (CGGTGTGTGCGGTGTCTGTAGT).

### Glycogen Measurement

Glycogen was purified and assayed as previously described [19–22]. 250–300 mg of skeletal muscle was weighed and dissolved in boiling 30% KOH solution to extract glycogen. To quantify glycogen levels, the product was digested with amyoglucosidase, and UV absorbance at 320 nm was measured after the addition of hexokinase.

## Results

### CETP expression does not alter exercise tolerance in chow-fed female mice

**Fig 1:**
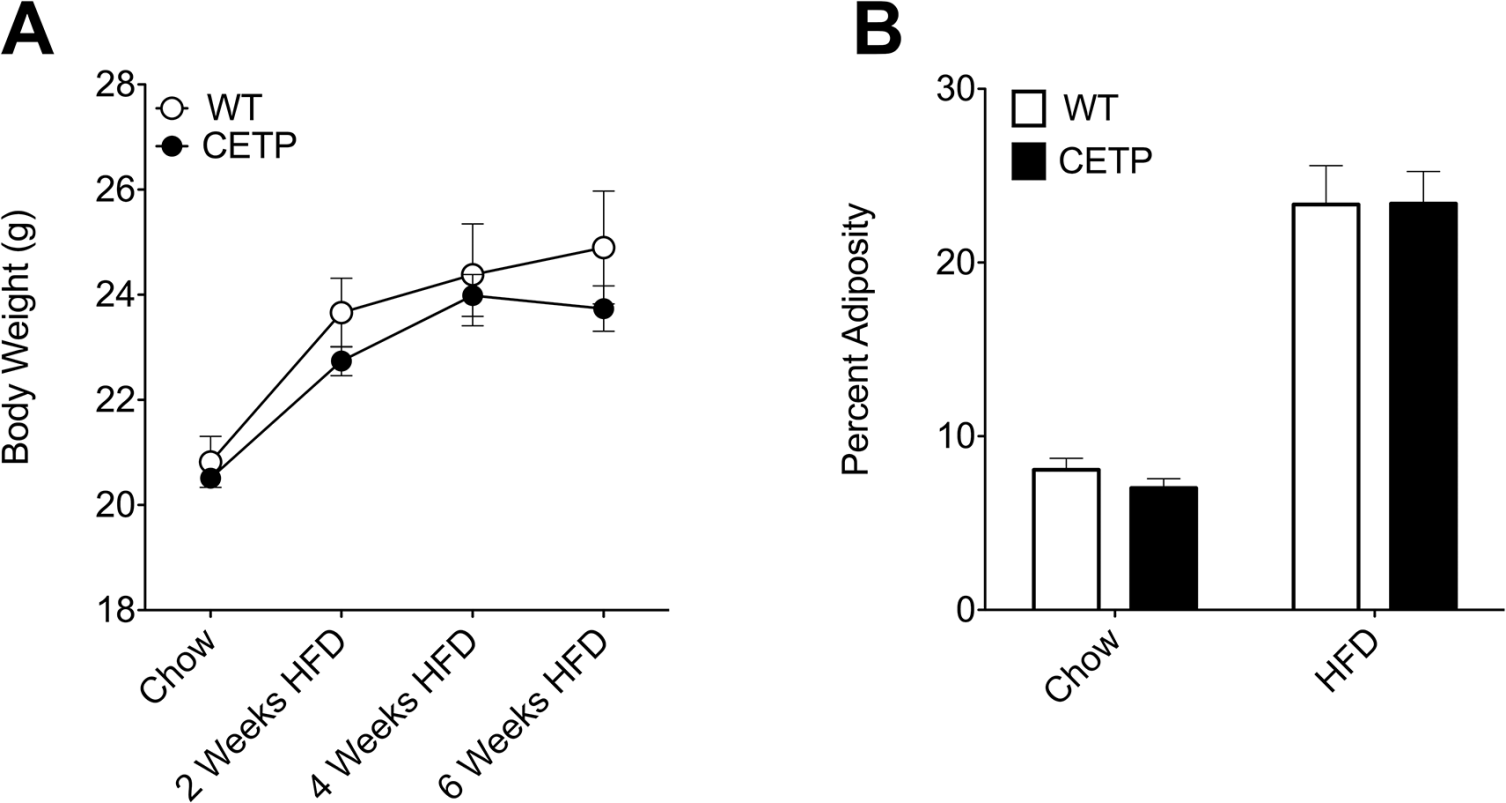
CETP expression does not alter weight or adiposity gain on HFD. (A) Body weight over the course of HFD-feeding. (B) Body composition at baseline and 4-weeks post HFD. n = 5–8 mice per group.

**Fig 2:**
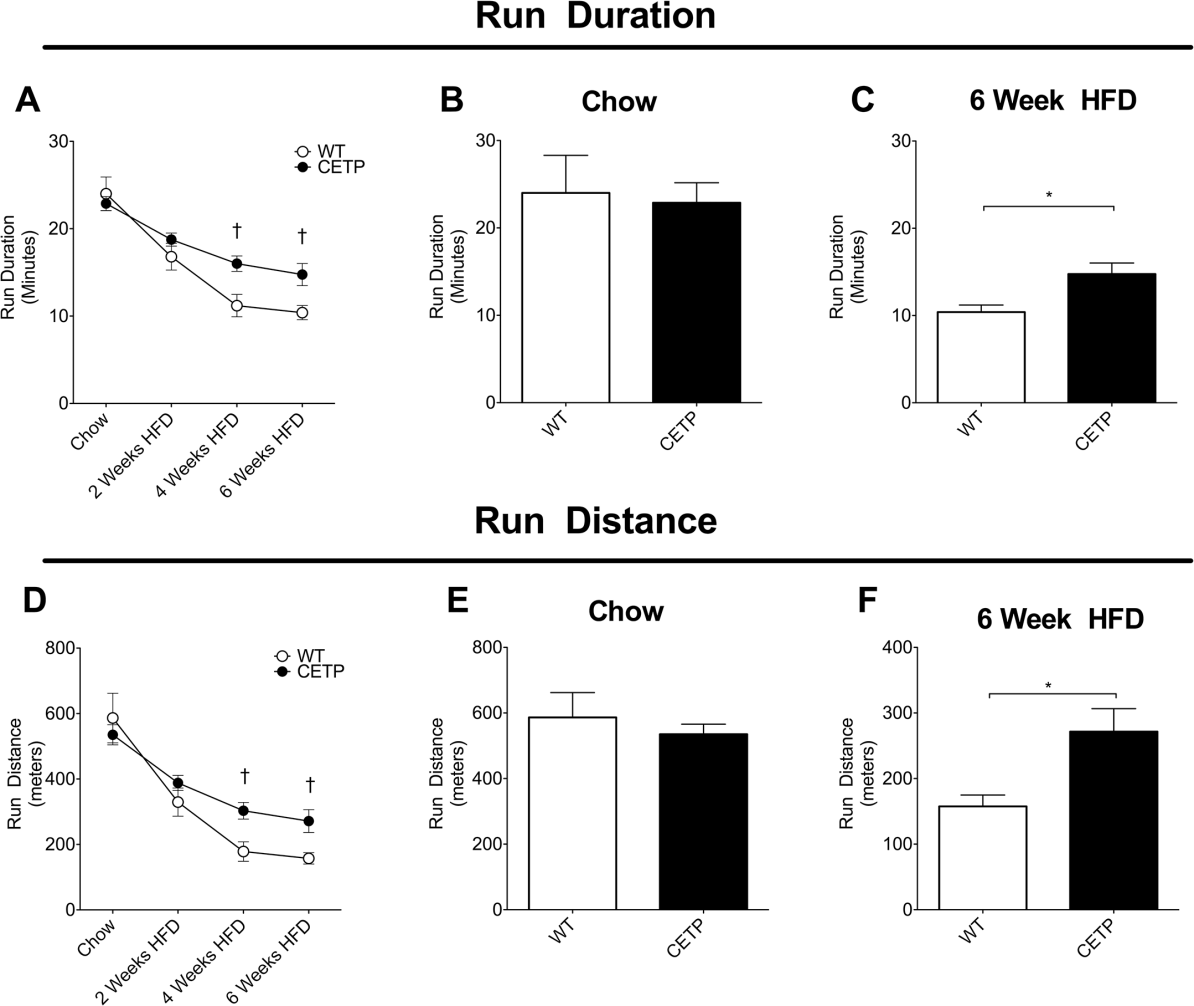
CETP expression improves endurance performance in female mice. (A) Run duration throughout the course of HFD. (B) Run duration in chow-fed mice before switch to HFD. (C) Run duration after 6 weeks of HFD-feeding. (D) Run distance throughout the course of HFD. (E) Run distance in chow-fed mice before switch to HFD. (F) Run duration after 6 weeks of HFD-feeding. Error bars represent mean ± SEM. n = 5–8 mice per group. † indicates significance by repeated measures ANOVA with Fisher’s least significant difference test. * indicates p<0.05 by unpaired t-test.

To test the effect of CETP expression on exercise tolerance in female mice, we performed an exercise study in CETP-expressing female mice and their non-transgenic wild-type female littermates. At the beginning of the study, mice were 12 weeks old, had been fed a standard chow diet since weaning, and were lean. There were no differences in initial body weight or body composition between genotypes (Fig 1A and 1B). Mice were subjected to an initial exercise tolerance test consisting of a treadmill run with speed increasing every 3 minutes. The duration, distance, and maximum work rate run by the mice provide an index of exercise capacity. In the initial exercise tolerance test, we saw no difference in exercise capacity between lean, chow-fed CETP mice and their WT littermates. CETP mice ran for an average of 23 ± 2 minutes (535 ± 90 meters) versus 24 ± 2 minutes (586 ±170 meters) for WT mice (Fig 2A, 2B, 2D and 2E). These results show that there is no genotype effect of CETP expression on exercise capacity in the context of a low-fat chow diet.

### CETP expression protects female mice from high fat diet-induced decline in exercise capacity

Because obesity induces a decline in exercise capacity, we measured the changes in exercise capacity in CETP-expressing female mice and their non-transgenic littermates over a period of HFD-feeding. Following the initial exercise test on chow diet, mice were switched to a 60% HFD to induce obesity. We have previously published that there are no significant differences in plasma fatty acid levels, liver TG content, serum estradiol, or plasma cytokines (IL-6, leptin, adiponectin) with CETP expression compared to WT in female mice in response to HFD-feeding [10]. Exercise capacity was measured every 2 weeks for 6 weeks using the same exercise tolerance test protocol used in the initial measurement. HFD-feeding effectively induced weight gain and increased adiposity in the mice. By 4 weeks on HFD, body weight increased over 15% in both groups of mice and both groups showed similar increases in adiposity (Fig 1A and 1B). The CETP-expressing mice were relatively protected against the decline in exercise capacity caused by obesity. At baseline, the CETP and WT mice ran for similar durations, 24 ± 2 minutes for WT and 23 ± 2 minutes for CETP. After 6 weeks of HFD, the CETP mice ran for 15 ± 4 minutes, significantly longer than the 10 ± 2 minute run duration seen in the WT (Fig 2A–2C). The run distance of the CETP mice (272 ± 99 meters) was also significantly longer after 6 weeks on HFD compared to the WT mice (158 ± 39 meters) (Fig 2D–2F). By the end of the 6-week course of HFD, the CETP-expressing mice ran 1.7-times the distance of the WT mice, demonstrating their relative protection against HFD-induced exercise intolerance (Fig 2F).

### CETP expression does not alter maximum oxygen consumption in exercising mice

**Fig 3:**
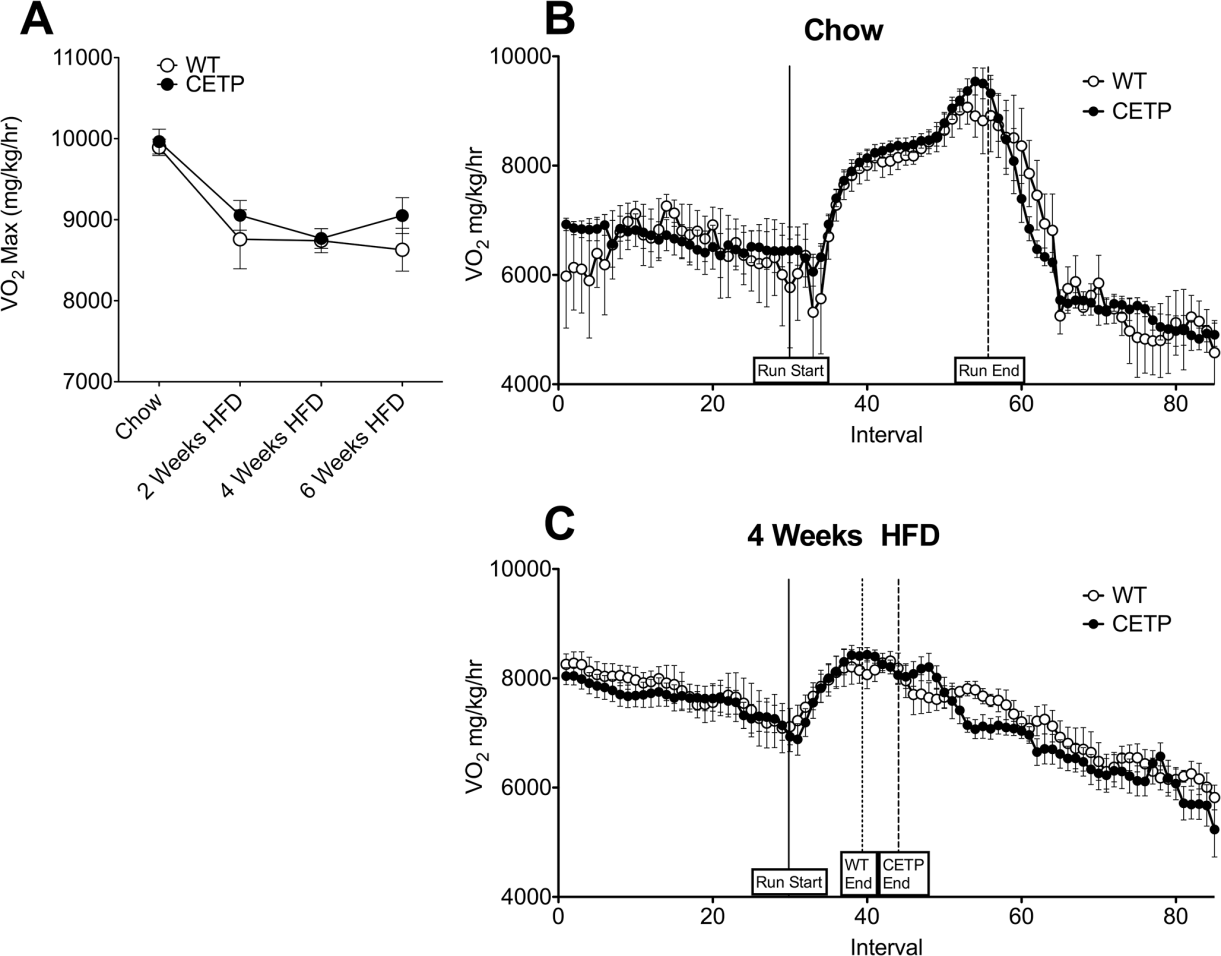
CETP expression does not alter VO_2_Max. (A) VO_2_Max in WT and CETP mice throughout the course of HFD. (B) Oxygen consumption in chow-fed mice during the initial exercise study. Solid line indicates beginning of exercise trial. Dashed line indicates median exercise time for both WT and CETP mice. (C) Oxygen consumption in HFD-fed mice during the exercise trial following 4 weeks of HFD. Solid line indicates beginning of exercise trial. Dotted line indicates median exercise time for WT mice. Dashed line indicates median exercise time for CETP mice. Error bars represent mean ± SEM. n = 5–8 mice per group.

Maximal rate of oxygen consumption during exercise (VO_2_Max) provides an index of aerobic physical fitness. To determine if CETP expression altered VO_2_Max, we measured the rate of oxygen consumption during the exercise tests performed every two weeks. There was no difference in VO_2_Max between lean, chow-fed WT and CETP animals in the first exercise test (Fig 3A). During the exercise test, VO_2_ increased from the beginning of the exercise bout (solid line) to the end of exercise (mean end time indicated by dashed line) in both groups. HFD impaired VO_2_Max as reflected by a 10% decline after 4 weeks relative to the chow-fed baseline (Fig 3A). When fed HFD, VO_2_ increased from the beginning of the exercise bout (solid line) to the end of exercise (mean end time indicated by dashed line for CETP and dotted line for WT) in both groups, but overall to a lesser extent than when the mice were chow-fed (Fig 3C). While aerobic capacity as measured by VO_2_Max is an important metric of exercise capacity, studies in sedentary humans suggest that exercise capacity in untrained individuals is limited by mitochondrial capacity and not aerobic capacity [23]. Therefore, we sought to assess mitochondrial capacity in the CETP mice.

### CETP-expressing mice show increased muscle oxidative capacity

**Fig 4:**
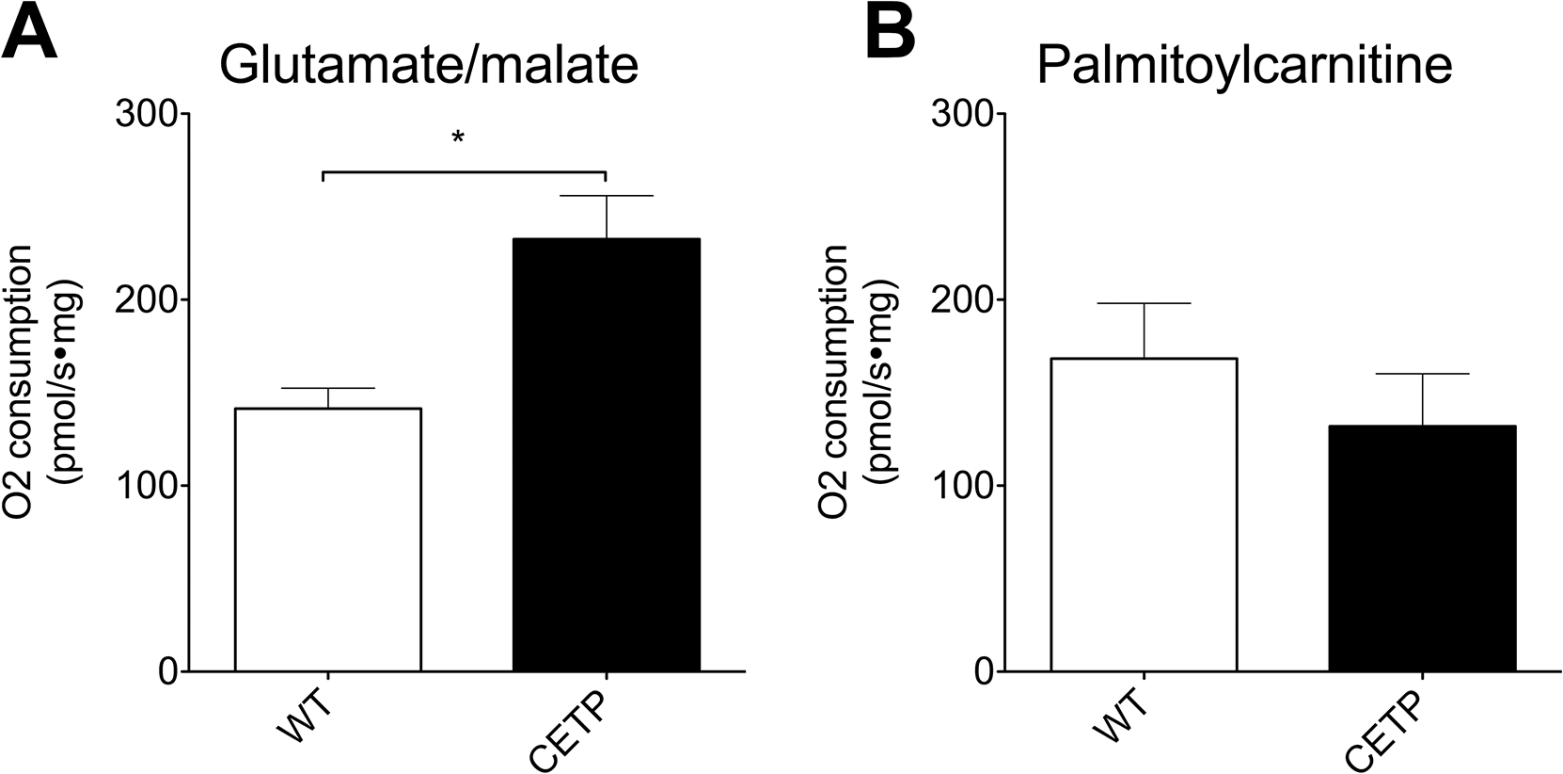
CETP expression increases mitochondrial oxidation in female mice. (A) Oxygen consumption in isolated muscle fibers treated with glutamate/malate mixture to measure total substrate oxidation. (B) Oxygen consumption in isolated muscle fibers treated with palmitoylcarnitine to measure fatty acid oxidation. Data represent mean ± SEM. n = 8–14 muscle fibers per group. * indicates p<0.05 by unpaired t-test.

To define the mechanism for CETP-mediated improvement in exercise capacity we examined muscle mitochondrial function after a final exercise bout following 12 weeks of HFD-feeding. To ensure equal exercise duration, both groups were subjected to a single 15-minute bout of exercise at a constant speed of 16 m/min. Muscle fibers from the red gastrocnemius muscle were isolated immediately following exercise, and mitochondrial oxidative capacity in these muscles was determined. Muscle fibers were treated with either glutamate-malate or palmitoylcarnitine to measure the response to TCA cycle intermediates or ATP production from fatty acid oxidation, respectively. We observed a significant increase in oxygen consumption in the CETP-derived muscle fibers when treated with the glutamate/malate mixture (Fig 4A) but no difference in oxygen consumption using the palmitoylcarnitine mixture (Fig 4B).

We also measured skeletal muscle glycogen levels in a cohort of ad-lib fed *vs*. post-exercise mice (CETP and WT). CETP and WT mice had similar levels of skeletal muscle glycogen in the fed state (CETP 37±15 mmol glc/kg tissue *vs*. WT 28±11 mmol glc/kg tissue). In response to exercise, CETP mice tended to have a greater decrease in glycogen content, though this result was not statistically significant between genotypes by ANOVA (CETP 4.2±4.4 mmol glc/kg tissue, a difference of 33 mmol glc/kg tissue from the fed state *vs*. WT 8.8±7.6 mmol glc/kg tissue, a difference of 19 mmol glc/kg tissue from the fed state).

Overall these results suggest that the mechanism for the improved exercise capacity is an increased ability of muscle to oxidize carbohydrate in the CETP-expressing mice. This is consistent with our prior observations that CETP-expressing female mice show increased muscle glycolysis defined by metabolite analysis and increased utilization of carbohydrate as a fuel source determined by calorimetry [10].

### CETP-expressing mice show increased gene expression for a mediator of mitochondrial function

**Fig 5:**
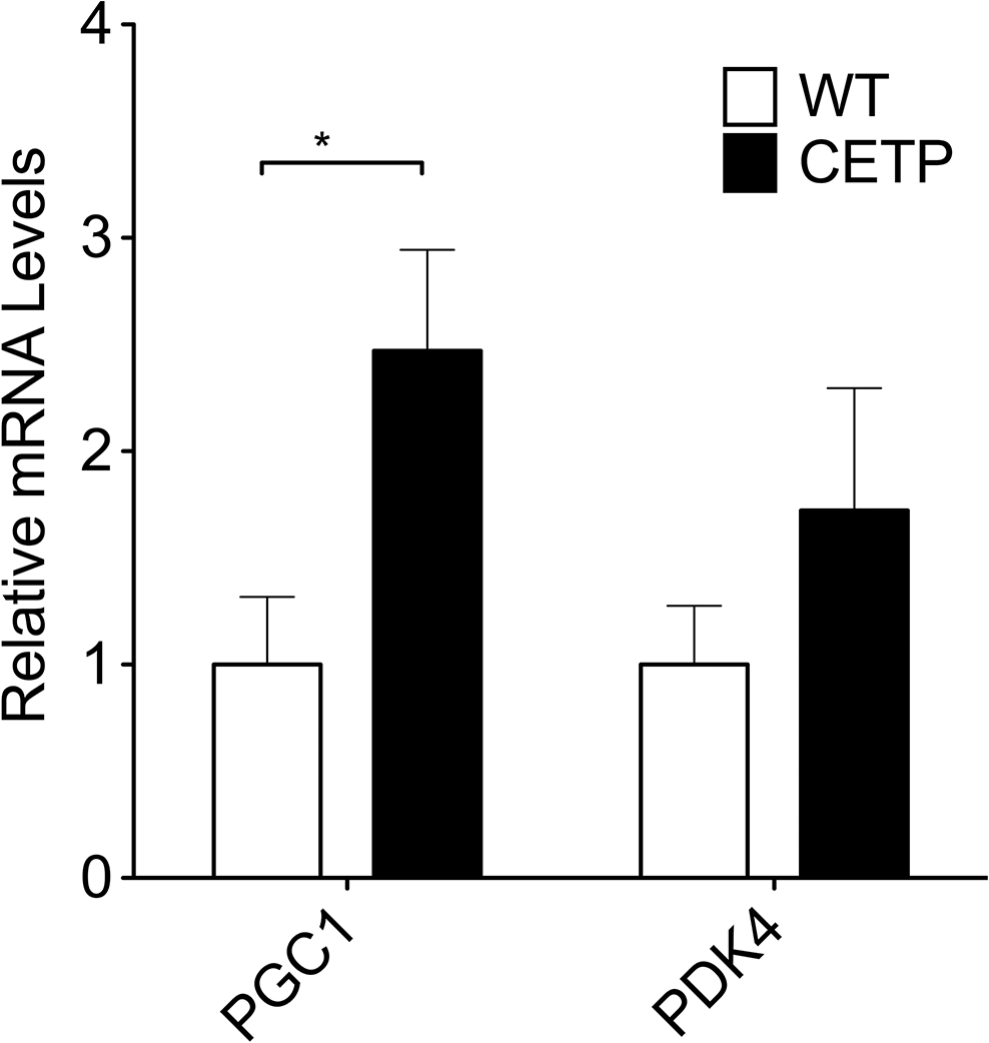
CETP expression increases mRNA for muscle PGC-1α. Gene expression in muscle tissue collected immediately following exercise. Data represent mean ± SEM. n = 4 mice per group. * indicates p<0.05 by unpaired t-test.

To relate whole-body exercise capacity and mitochondrial function to the molecular mediators of mitochondrial function, we measured mRNA for genes known to be changed by exercise, peroxisome proliferator-activated receptor gamma coactivator 1-alpha (PGC-1α) and pyruvate dehydrogenase kinase 4 (PDK4). PGC-1α is a transcriptional cofactor that is known to regulate mitochondrial function and is upregulated by exercise [24]. PDK4 is an enzyme that serves as a negative regulator of pyruvate dehydrogenase that is known to be upregulated following prolonged exercise [25, 26]. We observed a significant increase in mRNA for PGC-1α in the muscle of CETP mice compared to WT (Fig 5). There was no significant difference in PDK4 between CETP and WT (Fig 5).

## Discussion

Exercise capacity is an important index of human health. Impaired exercise capacity is a better predictor of mortality than increased BMI [2, 27], and even a modest improvement in exercise capacity can reduce risk of CHD in obese patients even without a change in weight [1, 5, 8, 28]. Even non-vigorous exercise such as walking has been shown to reduce CHD risk in women [8]. Exercise training has a positive effect on the anti-inflammatory properties of high-density lipoprotein [9]. Therefore, strategies that can improve exercise capacity even in the context of obesity could help to reduce risk of CHD.

We observed that CETP-expressing female mice were protected against HFD-induced exercise intolerance compared to WT. CETP expression resulted in an increase in intrinsic exercise capacity in obese, sedentary female mice. We previously observed that CETP-expressing female mice have increased muscle glucose flux to the TCA cycle and a preferential oxidation of carbohydrate compared to fatty acid [10]. The improvement in exercise capacity with HFD-feeding in this study corresponds with increased mitochondrial oxidative capacity of permeabilized muscle fibers in the CETP female mice compared to WT littermates. It is generally accepted that carbohydrate feeding increases exercise performance in athletes [29–31]. Increasing glucose delivery to muscle increases exercise capacity in sedentary mice [32]. Exercise training increases expression of GLUT-4 transporters in humans [33].

Increased muscle PGC-1α may link the metabolic improvements that we have previously observed in CETP mice to the improvement in exercise capacity seen in this study [10]. Mice that overexpress PGC-1α demonstrate increased substrate oxidation in isolated muscle mitochondria treated with malate and pyruvate [34]. We observed similar changes in substrate oxidation it the muscle of the CETP transgenic mice. Levels of PGC-1α mRNA are decreased in a mouse model of diet-induced obesity [35]. Reciprocally exercise training increases PGC-1α expression [24]. The increased oxidation of TCA cycle substrate in the muscle fibers of the CETP mice, the increased levels of PGC-1α mRNA, and our previous observation of increased carbohydrate utilization together suggest that the increase in exercise capacity observed with CETP expression is linked to improved mitochondrial carbohydrate oxidation, possibly by induction of PGC-1α.

Reverse cholesterol transport (RCT) is the process by which cholesterol is delivered to the liver and converted to bile for excretion into the gut. CETP plays a significant role in the process of RCT [36, 37]. CETP expression increases RCT and efflux of cholesterol to feces in the form of bile acids [10, 36]. Our findings are similar to the improvements in exercise capacity and mitochondrial oxidation observed in a mouse model of increased HDL cholesterol levels generated by ApoA1 overexpression [38]. ApoA1 is the scaffold protein of HDL, which promotes RCT. Our results suggest that high levels of HDL *per se* are not required for improved exercise capacity, as CETP expression in mice results in decreased serum HDL cholesterol levels, but increased RCT [11]. Improved cholesteryl ester delivery to the liver, and subsequently increased bile acid signaling may be responsible for the increased exercise capacity, which would be shared with both ApoA1 overexpression and CETP expression.

The results herein demonstrate that CETP expression protects female mice from the obesity-related decline in exercise capacity. This improvement in exercise capacity corresponds with improved muscle glucose oxidative capacity. CETP activity varies as much as 6–8 fold in human studies [39, 40]. Cholesterol feeding, insulin and ovarian hormones, all lead to significant changes in CETP activity [11, 41–44]. CETP activity and liver CETP gene expression are induced in with obesity in men and women [45, 46]. However, CETP activity is lower in patients who progress to diabetes [47–49]. Thus because CETP has a large natural variability, and declines with progression to diabetes, understanding how this pathway may be augmented to improve exercise capacity with obesity may be a new therapeutic opportunity.
